# Absence of Synaptic Regulation by Phosducin in Retinal Slices

**DOI:** 10.1371/journal.pone.0083970

**Published:** 2013-12-20

**Authors:** James H. Long, Vadim Y. Arshavsky, Marie E. Burns

**Affiliations:** 1 Center for Neuroscience University of California Davis, Davis, California, United States of America; 2 Depts of Ophthalmology and Pharmacology, Duke University Eye Center; Durham, North Carolina, United States of America; 3 Depts. of Ophthalmology & Vision Science and Cell Biology and Human Anatomy, University of California Davis, Davis, California, United States of America; Oregon Health & Science University, United States of America

## Abstract

Phosducin is an abundant photoreceptor protein that binds G-protein βγ subunits and plays a role in modulating synaptic transmission at photoreceptor synapses under both dark-adapted and light-adapted conditions in vivo. To examine the role of phosducin at the rod-to-rod bipolar cell (RBC) synapse, we used whole-cell voltage clamp recordings to measure the light-evoked currents from both wild-type (*WT*) and phosducin knockout (*Pd^−/−^*) RBCs, in dark- and light-adapted retinal slices. *Pd^−/−^*RBCs showed smaller dim flash responses and steeper intensity-response relationships than *WT* RBCs, consistent with the smaller rod responses being selectively filtered out by the non-linear threshold at the rod-to-rod bipolar synapse. In addition, *Pd^−/−^* RBCs showed a marked delay in the onset of the light-evoked currents, similar to that of a *WT* response to an effectively dimmer flash. Comparison of the changes in flash sensitivity in the presence of steady adapting light revealed that *Pd^−/−^* RBCs desensitized less than *WT* RBCs to the same intensity. These results are quantitatively consistent with the smaller single photon responses of *Pd^−/−^* rods, owing to the known reduction in rod G-protein expression levels in this line. The absence of an additional synaptic phenotype in these experiments suggests that the function of phosducin at the photoreceptor synapse is abolished by the conditions of retinal slice recordings.

## Introduction

Phosducin is one of the most highly expressed proteins in photoreceptors, yet its specific function in rods and cones has remained elusive. In its dephosphorylated state, phosducin binds to βγ subunits of heterotrimeric G-proteins [1]–[4], which led to the hypothesis that phosducin adjusts the gain of phototransduction by dynamically reducing the amount of G-protein available to be activated during light adaptation. However, suction electrode recordings from rod outer segments lacking phosducin (*Pd^−/−^*) showed no detectable deficits in light adaptation [5]. Instead, rods were less sensitive and showed reduced amplification, owing to a small reduction in G-protein expression levels that was a secondary consequence of the loss of phosducin [5].

Phosducin is expressed not only in the outer segments of photoreceptors but also the inner segments and synaptic terminals [6]–[9]. These observations prompted Herrmann et al. [10] to use the electroretinogram (ERG) b-wave to estimate the responses of dark- and light-adapted On-bipolar cells in wild-type (*WT*) and *Pd^−/−^* mice. After taking into account the aforementioned decrease in sensitivity of *Pd^−/−^* rod outer segments [5], the b-wave intensity-response functions of *Pd^−/−^* mice were shifted by a factor of 2.7 toward brighter intensities, and exhibited less desensitization in steady light, than those of *WT* mice. These differences were observed in both rod and cone pathways and attributed to the effects of phosducin at the synaptic terminal of photoreceptors [10].

Rods release glutamate not only onto rod bipolar cells (RBCs), but also onto subsets of On- and Off- cone bipolar cells, and are electrically coupled to cones that do likewise [11]. To evaluate the functional consequences of loss of phosducin specifically at the rod-to RBC synapse, we used single cell recordings to measure light-evoked currents from RBCs in acute retinal slices of *WT* and *Pd^−/−^* mice under both dark- and light-adapted conditions. Surprisingly, the intensity-response functions of the *Pd^−/−^* RBCs were not right-shifted compared to those of *WT* RBCs, but instead were slightly more non-linear (steeper). In addition, the dim-flash responses of *Pd^−/−^* RBCs were significantly delayed relative to those of *WT* RBCs, and showed less amplitude reduction in the presence of steady light. These results are consistent with reduced *Pd^−/−^* rod sensitivity and a non-linear threshold for transmission at rod-to-RBC synapse in retinal slices, but not consistent with previous in vivo ERG results.

## Materials and Methods

### Ethics statement

Mice in this study were cared for and handled according to procedures specifically approved by the Institutional Animal Care and Use Committee at the University of California at Davis and in strict accordance with the recommendations in the Guide for the Care and Use of Laboratory Animals of the National Institutes of Health.

### Animals

All mice were bred and reared in 12-hour cyclic light. In some experiments, c57Bl/6 and *Pd^−/−^* mice [9] were bred to generate *Pd^+/−^* mice that were subsequently bred in order to generate *Pd^−/−^* and *WT* littermate controls. There were no differences in the RBC properties of *WT* littermate controls and c57Bl/6 mice obtained commercially (Charles River), nor in the *Pd^−/−^* RBCs of outbred or inbred strains (data not shown).

### Retinal dissection and sectioning

Adult mice (3–6 weeks of age) were dark-adapted overnight, killed by cervical dislocation and decapitation, and the retinas removed under infrared light. Retinas were stored in a light-tight container for up to 6 hours and perfused with oxygenated, bicarbonate-buffered Ames solution maintained at 32°C. Retinal slices were prepared under infrared light by embedding the retina in low-temperature gelling agar (Agarose type VII-A, Sigma; 3% w/v in HEPES-buffered Ames solution) at temperatures no higher than 36°C. The agarose-embedded retina was bathed in HEPES-buffered Ames solution kept at 4°C and cut into 300 µm-thick slices using a vibrating microtome (VT1000S, Leica).

### Whole cell RBC recordings

Electrophysiology experiments were performed in a dark room using a Nikon E600FN upright microscope surrounded by a light-tight enclosure. Slices were transferred from the holding chamber to the recording chamber and perfused with bicarbonate-buffered Ames media (adjusted to pH of 7.4 with NaOH and to 300 mOsM with NaCl), equilibrated with 5% CO_2_/95% O_2_ and maintained between 34–37°C with a resistive in-line heating element (Warner Instruments, Hamden, CT), at a flow rate of approximately 4 ml/min. Retinal slices were illuminated with infrared light (λ>920 nm) and visualized using differential interference contrast (IR-DIC) optics. RBC somas were targeted based on their morphology and location within the distal portion of the inner nuclear layer. Borosilicate glass pipettes had electrical resistances between 10 and 15 MΩ in the bath when filled internal solution containing (in mM): 125 K-gluconate, 10 KCl, 10 HEPES, 0.1 CaCl_2_, 10 EGTA, 4 ATP-Mg, 0.4 GTP-Na_3_, and 0.1 Alexa-Flour 488 (Invitrogen, Carlsbad, CA). The electrode solution was adjusted to pH 7.2 with KOH and to between 285 and 290 mOsM with KCl. All chemicals were purchased from Sigma (St. Louis, MO) unless otherwise indicated.

Whole-cell voltage clamp recordings were made using an Axoclamp 200B amplifier (Axon Instruments, Forester City, CA). Signals were filtered using an eight-pole low-pass Bessel filter with a 200 Hz corner frequency and digitized at 5 kHz using a Digidata 1322A (Axon Instruments). Data were acquired using pClamp 6.0 software (Axon Instruments) and analyzed with a personal computer using custom software written with IgorPro (Wavemetrics, Lake Oswego, OR). Holding potentials were −60 mV, and the identity of some recorded cells were confirmed anatomically after the recording session by fluorescence microscopy. RBCs with maximal response amplitudes of less than 60 pA were discarded.

### Rod outer segment recordings

Suction electrode recordings from rod outer segments were as previously described [5], except that Ames media, rather than bicarbonate-buffered Locke's, was used to perfuse the tissue in the chamber and to fill the electrodes in order to match the recording conditions with those used for retina slice recordings. Because the electrode solution could not be continuously oxygenated as the bath perfusion was, the internal solution was supplemented with 10 mM HEPES, pH 7.4. All recordings were performed between 36–37°C.

### Light stimuli

Two high-power Luxeon white light-emitting diodes (Thor Labs, Ne*WT*on, NJ) were controlled using pClamp 6.0 software and a custom-built constant current generator. Stimuli were projected through a 500 nm band pass filter (10 nm FWHM) and attenuated with a neutral density filter before being delivered to the microscope via a fiber optic light guide and focused through a long working distance 20x condensing objective into a 550 µm diameter spot at the bottom of the recording chamber. Light intensities were measured with a silicon photodiode (United Detector Technology, Baltimore, MD); intensities were converted into photoisomerizations (R*/rod) using a rod effective collecting area of 0.4 µm^2^. A series of 10 ms flashes of calibrated strength were presented to elicit responses that spanned the dynamic range of each rod bipolar cell. Each flash family took approximately 1 minute to record, and responses to each flash-strength were averaged together. Averaged responses (R) were scaled by the cell's maximal response amplitude (R_max_) and plotted versus the flash strength (I) that elicited each response. These points were fitted with the Hill equation:
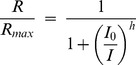
(Eq.1)where *I*_o_ is the half-saturating flash strength, and h is the Hill exponent.

For light adaptation experiments, steady light of calibrated intensity was presented for up to 10 seconds, after which time incremental flashes were delivered on top of this steady background. After the final flash, the steady light was extinguished and the cell was allowed to dark-adapt for up to 10 seconds before proceeding with additional flashes. Dark-adapted flash families were obtained before and after each light-adapted flash series and averaged together to correct for run-down that occurred during the course of the experiment; cells that did not show some degree of recovery were discarded and not used for light-adaptation analysis. In general, recordings in whole-cell configuration could be maintained for several minutes before rundown occurred. This constrained the duration of light adaptation experiments and often prevented data acquisition at more than one background intensity.

To further investigate maximal response amplitude suppression, dark-adapted rod bipolar cells were presented with a saturating flash in darkness. This flash was then repeated after 5 seconds of exposure to adapting background light with intensities that increased logarithmically from 0.25 to 750 photons/(µm^2^ s^−1^) over the course of the experiment. After every fourth flash the dark-adapted maximal response amplitudes were measured and used to correct for rundown that occurred during the course of the experiment.

### Modeling

The threshold-like non-linearity at the rod-to-RBC synapse was modeled as described [12]. Briefly, at each flash strength, a theoretical distribution of rod response amplitudes was generated, taking into account both the Poisson nature of photon absorption and the Gaussian dark noise caused by fluctuations of membrane current. For generation of the *WT* distribution, we assumed the SD of the dark noise was 0.27 pA, and the mean ± SD of the *WT* single-photon response was 1.0±0.33 pA [12]. For the *Pd^−/−^* distribution, the single-photon response mean and SD were both scaled according to the rod data recorded in Ames (0.9/1.19). These distributions were then transformed by a non-linear weighting function, such that rod responses with amplitudes smaller than the specified threshold were attenuated. The weighted distribution was then convolved with itself N times to simulate linear summation of the pooled rod inputs onto the RBC, and with a Gaussian distribution to represent noise intrinsic to the RBC. The dim-flash response (DFR) amplitudes of both *WT* and *Pd^−/−^* RBCs were accurately predicted by the model when the threshold was set to 0.9 pA and the number of rods converging onto a single RBC (*N*) was set to 30.

## Results

### Reduced dim flash sensitivity of rod bipolar cells in mice lacking phosducin

**Figure 1:**
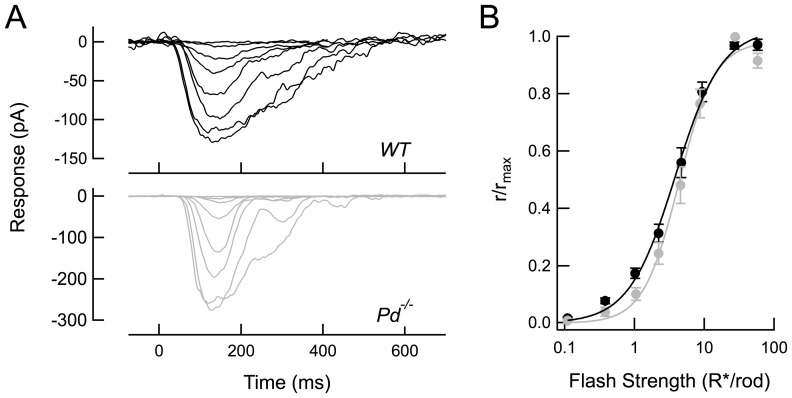
Dark-adapted RBC responses of *WT* and *Pd^−/−^* mice. (A) Representative families of responses to flashes that activated between 0.1 and 60 R*/rod. (B) Population average Intensity-Response (I-R) relationships from the averaged response amplitudes of 14 *WT* (black) and 11 *Pd^−/−^* (gray) dark-adapted RBCs. Error bars represent SEM. Curves were fitted using the Hill equation with Hill coefficients of 1.31 and 1.70 for *WT* and *Pd^−/−^*, respectively.

To investigate the role of phosducin at the rod-RBC synapse, we performed whole cell voltage-clamp recordings to measure the light-evoked currents of *WT* and *Pd^−/−^* RBCs. As expected, brief flashes of increasing strength produced responses of increasing amplitude, until the response amplitudes saturated (Fig. 1A). Responses were consistently evoked in *WT* and *Pd^−/−^* RBCs by flash strengths of 1 photon/µm^2^ and cells from both genotypes were generally driven to saturation by flash strengths of 75 photons/µm^2^, estimated to produce approximately 30 photoisomerizations per rod (R*/rod). The maximal response amplitudes (*r_max_*) of dark-adapted *WT* and *Pd^−/−^* RBCs were also not significantly different (Table 1; p = 0.65).

**Table 1:** Characteristics of dark-adapted rod bipolar cells recorded in Ames media.

	r_max_ (pA)	I_o_ (R*/rod)†	Non-linearity Factor††	Dim flash (r/r_max_)‡	Time to peak (ms)	S_f_^D^ (pA/R*/rod)†††
WT	−176±30 (14)	4.51±0.48 (14)	1.37±0.08 (14)	0.17±0.02 (14)	162±5 (14)	−27.3±4.6 (14)
Pd^−/−^	−160±21 (11)	5.06±0.53 (11)	1.95±0.11 (11)	0.10±0.02 (11)	156±4 (11)	−13.5±2.8 (11)

Values are mean ± SEM (number of cells).

† Flash strength that elicited a half-maximal response.

†† Hill coefficient describing the flash intensity-response relation.

‡ Amplitude of a response to a dim flash estimated to elicit approximately 1 R* per rod.

††† Dark-adapted sensitivity: flash response amplitude divided by flash strength.

To more closely examine the light-dependence of response amplitudes, the relationship between the averaged response amplitudes and flash strengths for each RBC were fitted with a Hill equation (Eq. 1). As reported previously, the RBC intensity-response (I-R) curves were best fitted with a Hill coefficient greater than 1, indicating a threshold-like non-linearity for transmission [12]. However, unlike the I-R comparisons of individual *WT* and *Pd^−/−^* rod outer segments in Locke's solution [5] and the I-R functions of *WT* and *Pd^−/−^* ERG b-waves [10], the half-saturating flash strengths for *WT* and *Pd^−/−^* RBCs were not significantly different (Table 1; p = 0.53). Instead, the I-R curves of *Pd^−/−^* RBCs were steeper than those of *WT* RBCs, requiring the fitted curves to have average Hill exponents that were 1.4-fold greater than those of *WT* (Table 1; p = 0.0002; Fig. 1B).

**Figure 2:**
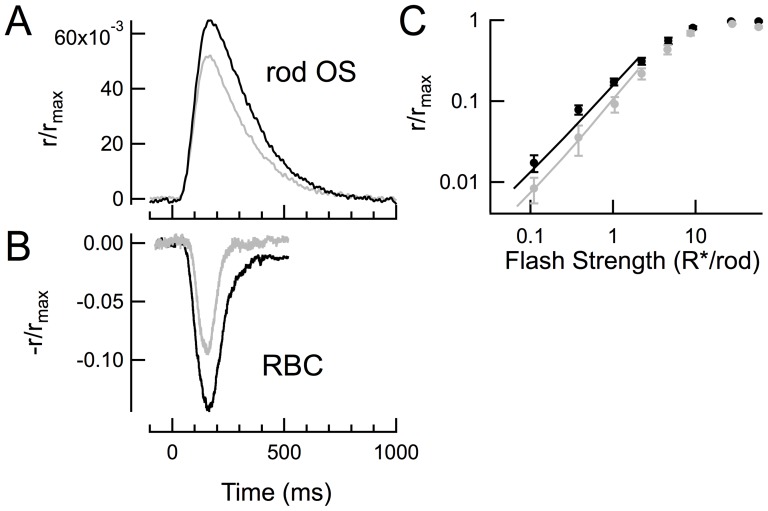
Smaller rod single photon responses can account for the steeper intensity-response relation of *Pd^−/−^* RBCs. (A) Mean normalized SPR amplitudes from 21 *WT* rods (black) and 17 *Pd^−/−^* rods (gray). Average dark currents for these populations were 15.9 and 16.4 pA, respectively. (B) Mean normalized dim flash responses estimated to elicit 1 R*/rod from 14 *WT* and 11 *Pd^−/−^* RBCs (see Table 1). (C) Predicted intensity-response relations for RBCs (solid lines) based on a model of the non-linear threshold that attenuates rod responses smaller than 1.1 pA (90% of the *WT* SPR amplitude). Points are the experimentally observed values, re-plotted from Fig.1.

The steeper I-R relationships of *Pd^−/−^* RBCs were caused primarily by smaller than normal responses to flash strengths that activated fewer than 3 R*/rod. The dark-adapted flash sensitivity (*S_f_^D^*; dim flash response amplitude divided by the flash strength) of *Pd^−/−^* RBCs was on average 2-fold lower than that of *WT* RBCs (Table 1; p = 0.007). The reduction in dim-flash sensitivity was also apparent in directly comparing the population average responses to a 2.5 photon/µm^2^ flash that activated approximately 1 R*/rod. At this flash strength, the normalized response amplitudes of *Pd^−/−^* RBCs were 41% smaller than *WT* controls (Table 1, Dim flash r/r_max_; p = 0.02; see also Fig. 2B). The reduction in dim flash response amplitudes is qualitatively consistent with the reduced G protein expression and the smaller single photon responses generated in *Pd^−/−^* rod outer segments [5].

### Compensation of reduced rod sensitivity at the rod-to-RBC synapse

The similarity of the half-saturating flash strengths of *WT* and *Pd^−/−^* RBCs suggests that the rod response “amplitude threshold” needed for reliable transmission across the synapse [12], [13] is unaffected by the loss of phosducin. Because *Pd^−/−^* rod responses are slightly smaller than those of *WT* rods, a similar amplitude threshold means that a greater fraction of *Pd^−/−^* rod single-photon responses should fail to be transmitted, resulting in smaller average RBC responses and a steeper intensity-response function. To determine whether the smaller average dim flash responses observed in *Pd^−/−^* RBCs are quantitatively consistent with the synaptic non-linearity, we used the model described by [12] to predict RBC response amplitudes from the *WT* and *Pd^−/−^* rod outer segment responses.

Because RBC responses are typically recorded in Ames media and rod outer segment recordings in Locke's, and because the outer segment responses in Ames and Locke's show striking differences in amplitude and kinetics [14], [15], we performed suction electrode recordings from *WT* and *Pd^−/−^* rods in Ames media so that they could be directly compared with the RBC results. As previously described for *Pd^−/−^* rods in Locke's solution [5], *Pd^−/−^* rods were less sensitive, with a smaller average single photon response amplitude (Fig. 2A) and a larger half-saturating flash strength value than observed in *WT* rods (Table 2).

**Table 2:** Characteristics of dark-adapted WT and Pd^−/−^ rods recorded in Ames media.

	r_max_ (pA)	I_o_ (photons/μm^2^)†	SPR (pA)‡	Time to peak (ms)	Int time (ms)	Coll Area (μm^2^)	τ_D_ (ms)
WT	17.7±0.7 (23)	30.9±2.4 (23)	1.19±0.11 (21)	152±9 (23)	234±15 (23)	0.44±0.03 (21)	167±10 (23)
Pd^−/−^	16.9±0.7 (23)	45.0±3.2 (23)	0.90±0.09 (17)	142±9 (23)	230±12 (23)	0.43±0.05 (17)	169±8 (23)

Values are mean ± SEM (number of cells).

† Flash strength that elicited a half-maximal response; p = 0.0007.

‡ Single photon response amplitude; p = 0.045.

For all others, p≥0.38.

In predicting the RBC response amplitudes from the rod outer segment responses, there are two important variables: the value of the amplitude threshold (the rod response amplitude below which rod responses are lost), and the number of rods that converge on a given RBC. Assuming an amplitude threshold of 0.9 pA and a convergence of 30 rods for both mouse strains, the non-linear threshold model accurately predicted the experimentally observed RBC intensity-response curves (Fig. 2C) for *WT* RBCs. Using these same parameters and only changing the average and SD of the *Pd^−/−^* rod single-photon response amplitudes (Table 2), the model produced a steeper intensity response curve (Fig. 2C, straight lines) that accurately matched the experimental data (Fig. 2C, data points).

### Increased synaptic delay at the rod-to-RBC synapse of *Pd^−/−^* mice

**Figure 3:**
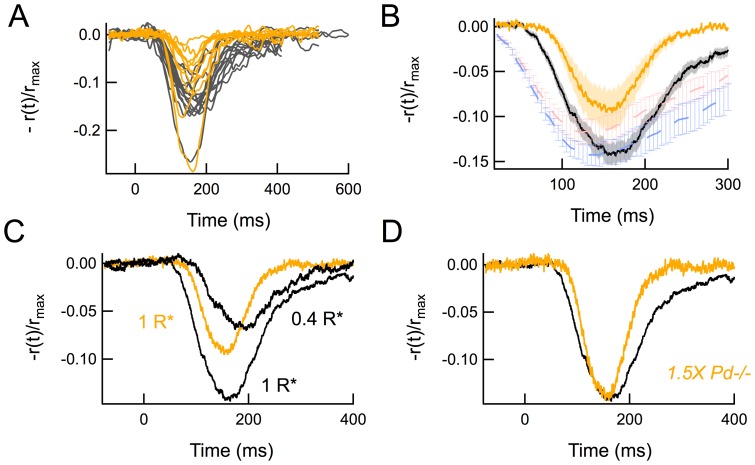
*Pd^−/−^* RBC responses show a delayed onset that is consistent with smaller average responses. (A) Average responses to dim flashes (approximately 2.5 photons/µm^2^) estimated to elicit 1 R*/rod from 14 *WT* RBCs (black) and 11 *Pd^−/−^* RBCs (yellow). Responses were normalized by each cell's maximal response amplitude and low-pass filtered at 50 Hz. (B) Average and standard errors of the traces in A. The population average rod single photon responses from Fig. 2 (*WT* rods blue, *Pd^−/−^* rods red) have been inverted, scaled and superimposed for temporal comparison. (C) Average RBC responses from B compared to the average *WT* RBC response to a dimmer flash (0.4 R*/rod). The *Pd^−/−^* synaptic delay and amplitude are intermediate to the two *WT* traces. (D) Average responses from B, with the *Pd^−/−^* RBC response (yellow) scaled by a factor of 1.5, providing a poor temporal match despite aligning peak amplitudes with the average *WT* RBC response (black).

Another notable feature of *Pd^−/−^* RBC responses was a pronounced temporal lag in the development of the inward current following the flash. All *Pd^−/−^* RBCs responses showed a greater synaptic delay than *WT* RBC responses to the same flash strength, despite the usual cell-to-cell fluctuations in response amplitudes (Fig. 3A). Superimposition of the population average responses (Fig. 3B) revealed the magnitude of this delay to be 21 ms, which coincided roughly with the earliest deviations of the rod outer segment responses between the two genotypes (red and blue traces, Fig. 3B).

We therefore wondered whether the increased synaptic delay in the *Pd^−/−^* RBC responses was again a result of the smaller than average response amplitudes and the non-linear amplitude threshold operating at this synapse. To test this idea, we compared the onset of the average *Pd^−/−^* response to a 1 R*/rod flash to an average *WT* response of comparable peak amplitude (0.4 R*/rod). Indeed, comparison of these two responses of similar peak amplitudes had very similar synaptic latencies (Fig. 3C). In contrast, merely scaling the *Pd^−/−^* response to match the peak amplitude of the *WT* response provided a very poor match (Fig. 3D), illustrating the consequences of the non-linear threshold not only on the amplitude but also the time course of the RBC response recorded in the slice preparation.

### Reduced desensitization of rod bipolar cells by steady light in *Pd^−/−^* mice

In previous ERG measurements, *Pd^−/−^* mice showed a reduced degree of b-wave desensitization in the presence of steady light, suggesting that photoreceptors lacking phosducin did not reduce their synaptic gain appropriately or behaved as if they were already light-adapted [10]. To determine whether RBCs in the *Pd^−/−^* retina show normal light adaptation under voltage clamp conditions, we recorded flash families of *WT* and *Pd^−/−^* RBCs in the presence of 128 photon/µm^2^/sec background light (estimated to produce approximately 50 R*/rod/sec). We chose this intensity because light-induced desensitization of the ERG b-wave is impaired at comparable backgrounds [10] and because less intense backgrounds have been shown to increase gain at individual rod-RBC synapses [16].

**Figure 4:**
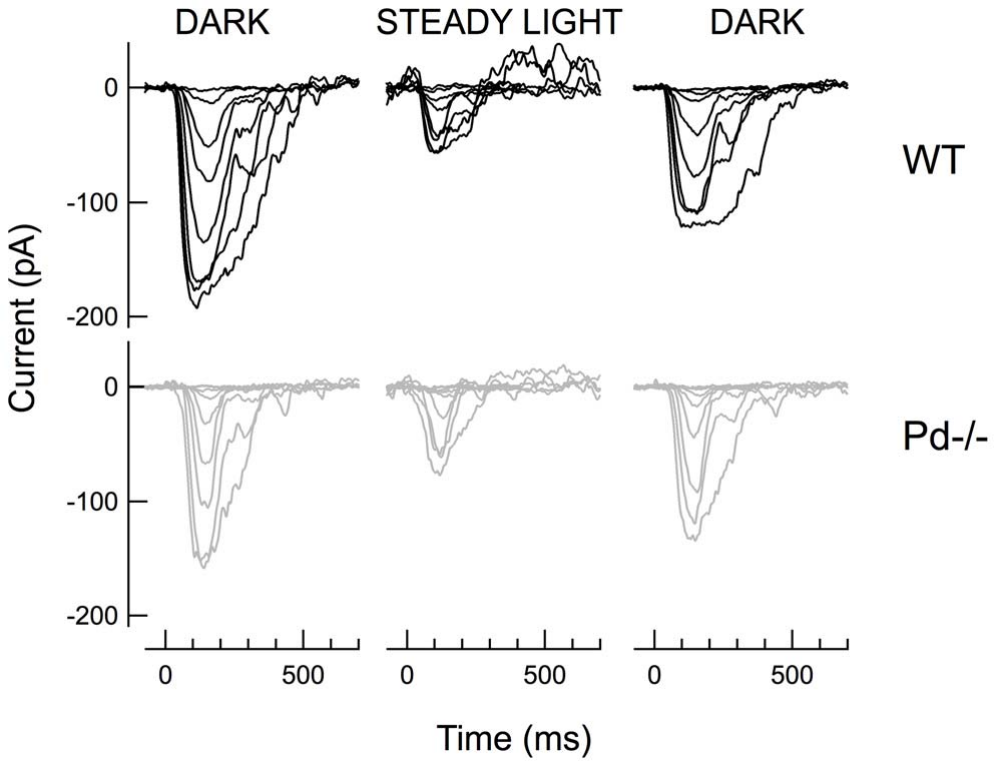
Families of dark- and light-adapted flash responses recorded from *WT* (black) and *Pd^−/−^* (gray) RBCs, before, during and after exposure to steady light estimated to elicit 51 R*/rod sec^−1^.

**Figure 5:**
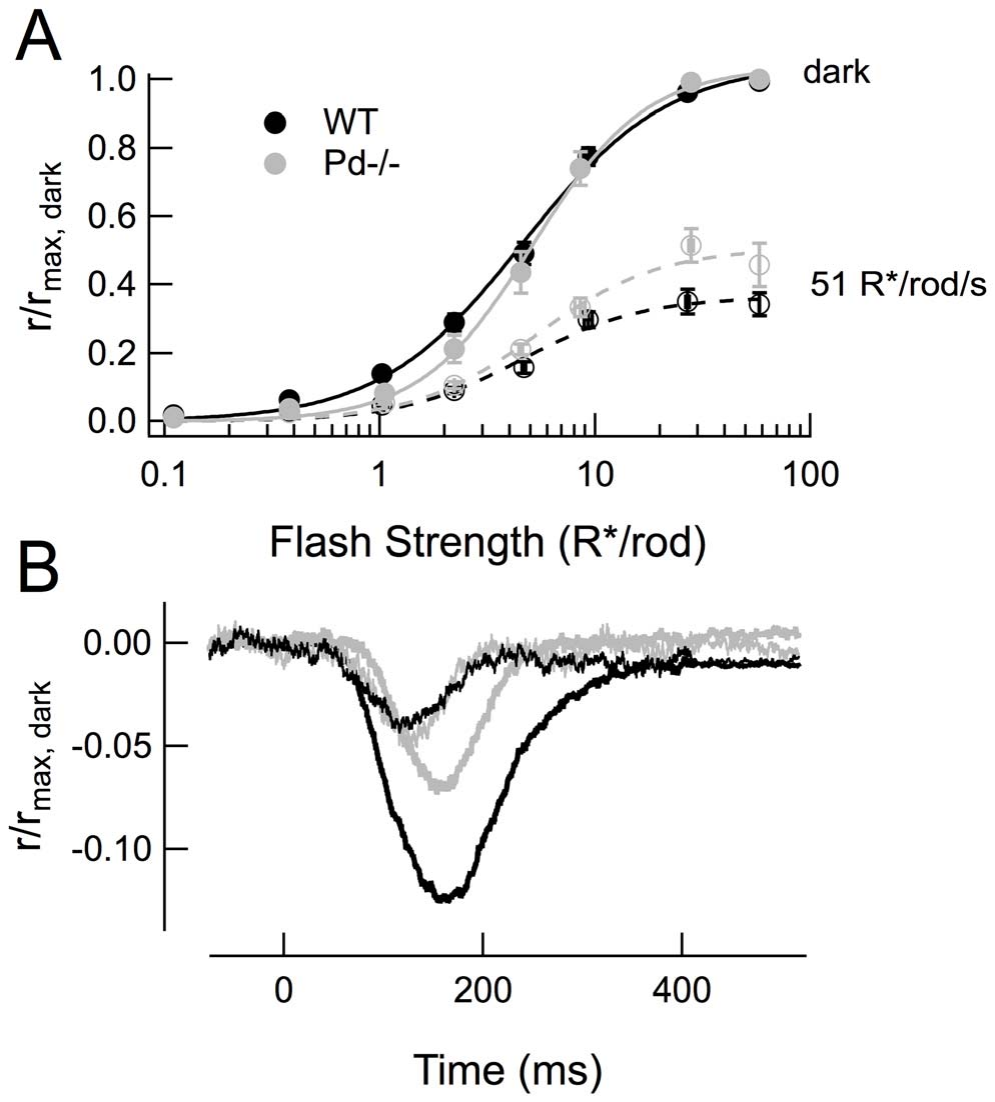
*Pd^−/−^* RBCs exhibit less reduction of dim-flash response amplitude and suppression of r_max_ than *WT* RBCs in the presence of background light. (A) Composite I-R relationships created by fitting Hill equations to the averaged response amplitudes ± SEM of 12 *WT* and 10 *Pd^−/−^* dark and light-adapted RBCs. Dark-adapted data points (filled symbols) were determined by averaging response amplitudes recorded before after recording light-adapted flash families in the presence of 51 R*/rod/sec background light (open symbols). (B) Averaged normalized responses of the same cells to a flash estimated to elicit approximately 1 R*/rod in the presence of the 51 R*/rod/sec background light.

In the presence of this background intensity, the response to a given flash strength was substantially smaller than the responses to the same flash before and after the steady background in both *WT* and *Pd^−/−^* RBCs (Fig. 4). However, neither of the intensity-response relationships for *WT* and *Pd^−/−^* RBCs was shifted to the right of the dark-adapted curves (*WT* p = 0.12; *Pd^−/−^* p = 0.97; Fig. 5A), unlike the previously observed light-adapted ERG b-wave measurements [10]. Likewise, during light adaptation, *WT* and *Pd^−/−^* responses became even more similar; there was no significant difference in the normalized amplitudes of dim flash responses in the presence of the background (p = 0.44; black and gray thin traces, Fig. 5B; Table 3), and thus no difference in the light adapted flash sensitivity (S_f_^L^; Table 3). However, *Pd^−/−^* RBCs did exhibit a smaller fractional change in flash sensitivity relative to their dark-adapted values, owing to the smaller S_f_^D^ discussed above (compare the difference in peak amplitudes between thick and thin traces, Fig. 5B; Table 3, S_f_^L^/S_f_^D^; p = 0.0008).

**Table 3:** Characteristics of WT and Pd^−/−^ RBCs in steady light activating 50 R* rod^−1^s^−1^.

	Suppression†	I_o_ (R*/rod)	Non-linearity Factor	Dim flash (r/r_max_)	Time to peak (ms)	S_f_^L^ (pA/R*/rod)†††	S_f_^L^/S_f_^D^ †††
WT	0.40±0.03 (12)	6.98±1.16 (12)	1.56±0.19 (12)	0.05±0.006 (12)	125±6 (12)	−5.06±1.28 (12)	0.25±0.04 (12)
Pd^−/−^	0.54±0.04 (10)	5.07±0.63 (8)	1.67±0.10 (8)	0.05±0.004 (10)	130±4 (10)	−5.07±0.70 (10)	0.68±0.11 (10)

Values are mean ± SEM (number of cells).

† Fractional reduction of the maximal response amplitude by steady light activating 50 R* rod^−1^s^−1^; p = 0.01.

††† Flash sensitivity: average response amplitudes divided by flash strength for flashes delivered in steady light (S**_f_** ^L^) or in darkness (S**_f_** ^D^).

Other parameters as defined in Table 1. For S**_f_** ^L^/S**_f_** ^D^ p = 0.0008; for all others not indicated, p≧0.23.

**Figure 6:**
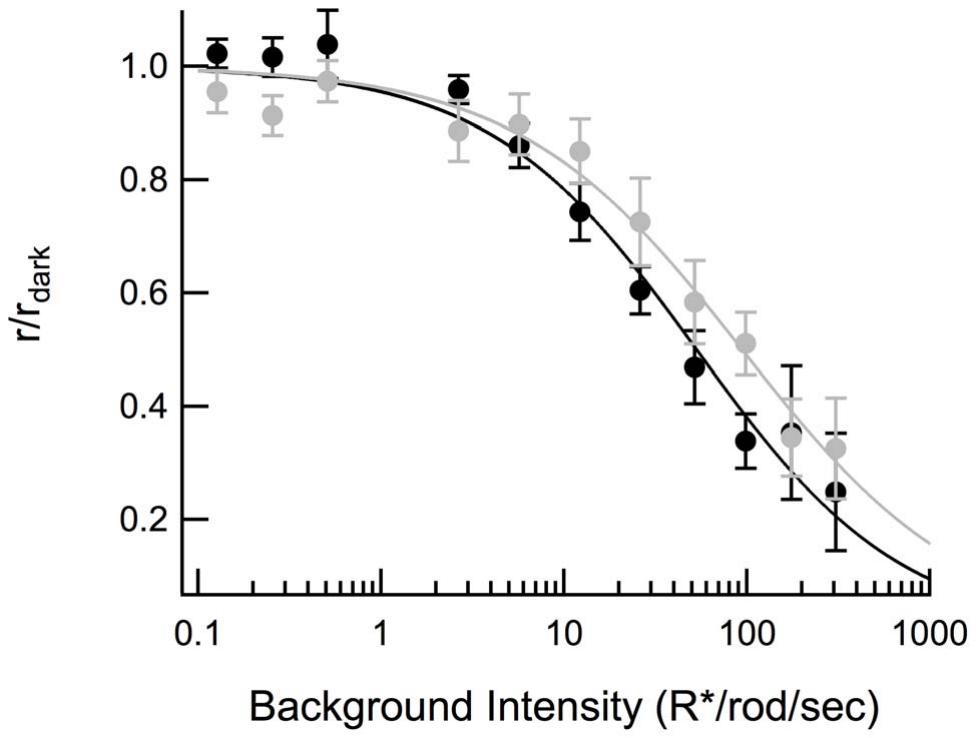
Suppression of maximal response amplitude by background light. Dark-adapted *WT* (black) and *Pd^−/−^* (gray) RBCs were presented with a saturating flash of 36 R*/rod and the same stimulus was subsequently presented after 5 seconds of exposure to background light that was increased logarithmically between 0.1 of 300 R*/rod/sec at the beginning of each trial. Curves were fitted using the Hill equation. The background intensity that suppressed half of the maximal dark-adapted response amplitude was 52 and 106 R*/rod/sec for *WT* and *Pd^−/−^*, respectively.

Background light reduced the maximal (saturating) flash response amplitudes of both *WT* and *Pd^−/−^* RBCs (Fig. 5A). However, the reduction of the maximal amplitude was less for *Pd^−/−^* RBCs than for *WT* RBCs (p = 0.01; Table 3), consistent with the smaller fractional change in flash sensitivity in the absence of phosducin. This reduced “desensitization” of the maximal response amplitude in steady light persisted across a wide range of background light intensities (Fig. 6). The steady light intensity at which the RBC maximal response was reduced by one-half (*I_o_*) was 2-fold greater for *Pd^−/−^* RBCs than for *WT* RBCs (Fig. 6; p = 0.04). Thus, light did not desensitize *Pd^−/−^* RBCs to the same extent that it desensitized *WT* RBCs, consistent with the reduced desensitization previously observed in *Pd^−/−^* rod outer segments during light adaptation [5].

### Effect of steady light on rod-RBC synaptic delay

**Figure 7:**
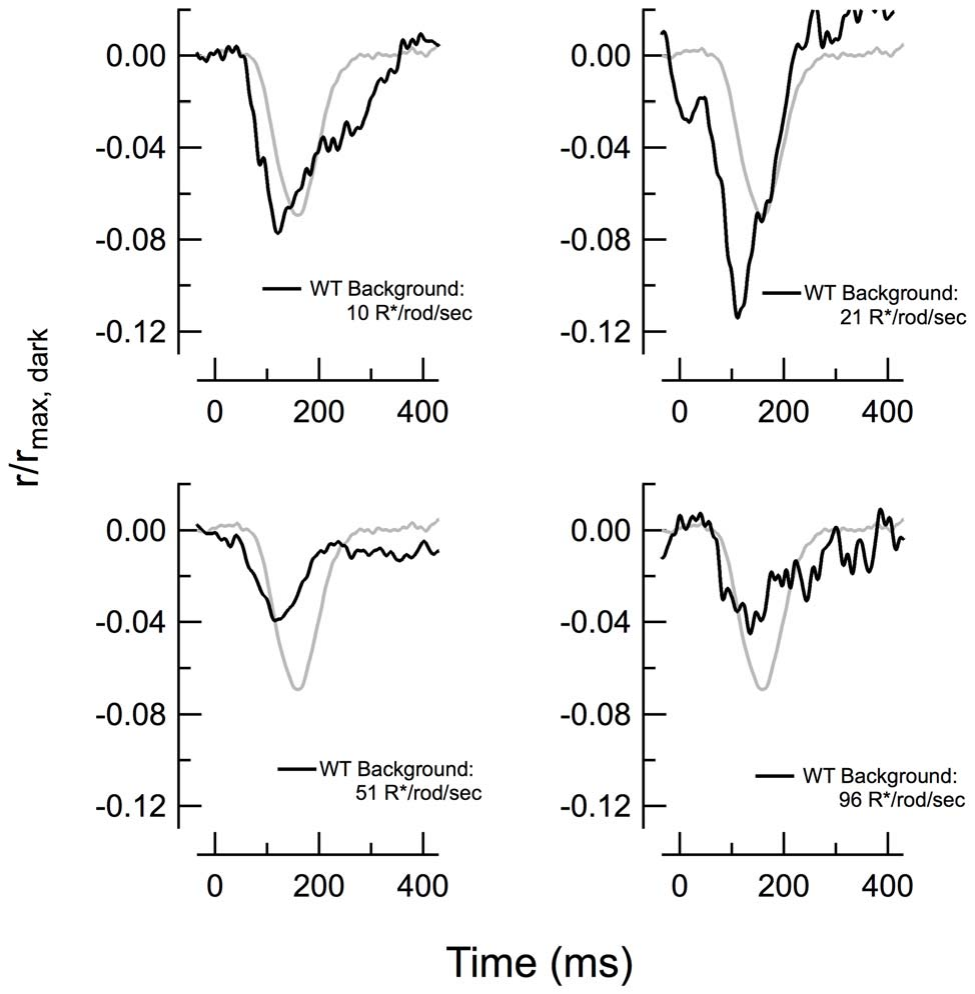
Light adaptation in normal retina has little effect on synaptic delay. Responses to flashes estimated to elicit 1 R*/rod were delivered to *WT* RBCs in the presence of steady background light of indicated intensities (black traces). The normalized average dark-adapted *Pd^−/−^* response to the same 1 R*/rod flash is shown for comparison in each panel (gray, from Fig. 2A).

Light adaptation in rods affects both rod response amplitude and kinetics and thus provides an additional tool by which to examine the relationship between incremental response amplitude and the synaptic delay (e.g. Fig. 3). We compared the consequences of steady light on the response onset of *WT* RBCs. Adapting *WT* retinas to dim light intensities first potentiated the RBC response amplitude (e.g. at 21 R*/rod/s) and then diminished it at higher backgrounds (e.g. 96 R*/rod/s; Fig. 7), as previously described [16]. However, none of these light intensities much altered the onset time of the light-adapted responses, suggesting that the baseline rate of glutamate release was unaffected by these backgrounds. In no instance did the form of the response resemble that of the average dark-adapted *Pd^−/−^* RBC response (gray trace in Fig. 7).

## Discussion

### Consequences of the loss of phosducin on the rod-to-RBC synapse

In this study, we set out to test the idea that the photoreceptor-specific protein, phosducin, alters synaptic transmission onto rod bipolar cells. Recordings from rod bipolar cells in retinal slices revealed four significant changes in *Pd^−/−^* rod transmission, compared to WT: 1) decreased dim flash RBC response amplitude; 2) steeper flash intensity-response relationship; 3) reduced desensitization in steady light; and 4) an increased synaptic delay.

All four findings appear to be inter-related and to stem from the reduced amplification and sensitivity of *Pd^−/−^* rod outer segments. We have shown that both the reduced amplitude of *Pd^−/−^* RBC dim flash responses and the steeper intensity-response relationship are quantitatively predicted by the difference in amplitudes of the *Pd^−/−^* rod single photon responses (Fig. 2). The reduced RBC desensitization in steady light (the need for 2-fold brighter light for equivalent adaptation; Fig. 6) is similar to the 1.5-fold reduction in sensitivity observed in *Pd^−/−^* rod outer segments (Table 2; [5]).

### Reduced rod sensitivity does not alter synaptic threshold or synaptic gain adjustment

One consequence of this study has been an examination of how changes in rod flash sensitivity affect transmission of information across the first synapse of the primary rod pathway. We have confirmed the reduction in rod flash sensitivity in *Pd^−/−^* rods, and have further shown that the smaller-amplitude responses result in greater rejection by the non-linear threshold at the rod-to-RBC synapse. As a result, RBCs display steeper than normal intensity-response relations in dark-adapted slices. These results support the notion that the threshold amplitude at this synapse is stationary, at least under voltage-clamp, and not determined dynamically through a feedback mechanism that can sense the amplitude of the elementary responses. This notion is also supported by previous work by Sampath and colleagues who demonstrated the converse: that a genetic perturbation that increases the rod's single-photon response amplitude does not alter the synaptic threshold and instead leads to a greater fraction of elementary events being relayed across the synapse, resulting in a shallower than normal intensity-response curve [13].

Unlike photoreceptors, which dramatically reduce their flash sensitivities in the presence of steady light, the RBCs in our study showed little reduction in sensitivity, even in backgrounds that produced sustained bleaching rates of up to ∼100 R*/rod/s. Instead, we observed an increase in relative flash sensitivity in dim backgrounds (21 R*/rod s^−1^; Fig. 7), consistent with previous studies [16]. Likewise, we saw little appreciable rightward shift of the intensity-response relations in steady light (Fig. 5A), consistent with other reports [17]. These general characteristics were not significantly different in RBCs of *Pd^−/−^* mice, suggesting that these characteristic changes in synaptic gain at the photoreceptor synapse in retinal slices are not regulated by phosducin.

### Reconciliation with in vivo recordings and future directions

In the original 2010 study of Herrmann et al. [10], the authors compared the ERG b-waves of *WT* mice to those of *Pd^−/−^* mice and found that the half-saturating flash strengths (I_o_) of dark-adapted rod-driven b-waves were shifted by a factor of 2.7 toward brighter intensities. Half of this effect was attributed to reduced phototransduction gain, suggesting that the other half originated from reduced glutamate release from photoreceptor synaptic terminals in the knockout. In contrast, we have found that single-cell recordings from dark-adapted *Pd^−/−^* RBCs in retinal slices show no significant loss of sensitivity that could be measured by a rightward shift in I_o_. Furthermore, the 2-fold rightward shift in the light intensities required to suppress the maximal response amplitude in our experiments (Fig. 6) is significantly less than the ∼11-fold rightward shift in a comparable adaptation protocol measured by ERG b-wave [10]. What explains these slice vs. in vivo disparities?

Let us first consider the smaller reduction in phototransduction gain in *Pd^−/−^* rods in the slice. Note that the previous comparison of phototransduction gains in *WT* and *Pd^−/−^* rods was performed in in Locke's solution [5], whereas here we compared responses of rods and RBCs of both genotypes in Ames media (Table 2) for consistency. Single photon response amplitudes of *WT* and *Pd^−/−^* rods are roughly 2-fold different when recorded in Locke's and only ∼25% different when recorded Ames (Table 2). In addition, the average *WT* single-photon response amplitudes in Ames are significantly larger than those in Locke's. These differences imply that outer segment responses in Ames are closer to local amplitude saturation, which will minimize the difference in synaptic release attributable to reduced phototransduction gain. As discussed in [14], recordings in Locke's may more closely represent the *in vivo* conditions of ERGs recordings. This would explain why the photoresponse amplitude reduction in *Pd^−/−^* rods documented in this study is smaller than those measured in single cell recordings in Locke's and in ERG experiments.

Our second observation that differs from ERG is the lack of evidence for any functional role for phosducin in the rod synaptic terminal. This phenotype suggests that the synaptic function of phosducin requires additional conditions or factors that are missing in the slice preparation. For example, all of the RBC recordings herein were made under voltage-clamp, which precludes postsynaptic detection of any phosducin-dependent presynaptic modulation that may alter the RBC resting potential. Another possibility is that phosducin in photoreceptor terminals requires neuromodulatory inputs that are not preserved in the slice. Finally, recordings from perfused retinal slices immersed in solution may not perfectly recapitulate natural ionic microenvironments of the cells in the outer retina.

The incongruence of phosducin knockout phenotypes observed by ERG vs. slice recordings is only one of a list of more general differences between results obtained by these techniques. It is well-known that the intensity-response functions of rod b-waves have Hill coefficients of ∼1, while the intensity-response functions of individual RBCs in slices are supra-linear. The supra-linearity observed in slices has long served as the conceptual framework for the basis of the non-linear threshold to selectively attenuate noise and small single-photon responses [12]. It is also thought to account for visually-guided behavior at visual threshold [13], making the disparity between measurements and methodologies important to reconcile. Both methods are imperfect: ERG b-waves, being field potentials, are robust but reflect complex summation of multiple cell types in the retina. Slice recordings permit subtype cell classification and more controlled light delivery, but are fragile and run-down rapidly. In addition, slices invariably lack full biological context of the retinal circuitry and endogenous composition of ionic and neurotransmitter gradients. Therefore, development of cell-type specific, in vivo measurements to examine the molecular underpinnings of synaptic regulation is an essential next step in retinal physiology.
